# Metagenomic Sequencing for Microbial DNA in Human Samples: Emerging Technological Advances

**DOI:** 10.3390/ijms23042181

**Published:** 2022-02-16

**Authors:** Yu Shi, Guoping Wang, Harry Cheuk-Hay Lau, Jun Yu

**Affiliations:** Institute of Digestive Disease, Department of Medicine and Therapeutics, State Key Laboratory of Digestive Disease, Li Ka Shing Institute of Health Sciences, CUHK Shenzhen Research Institute, The Chinese University of Hong Kong, Hong Kong, China; shiyu2019@link.cuhk.edu.hk (Y.S.); wangguoping@link.cuhk.edu.hk (G.W.); harrylau@link.cuhk.edu.hk (H.C.-H.L.)

**Keywords:** high throughput sequencing, human microbiome, host DNA depletion, clinical metagenomics

## Abstract

Whole genome metagenomic sequencing is a powerful platform enabling the simultaneous identification of all genes from entirely different kingdoms of organisms in a complex sample. This technology has revolutionised multiple areas from microbiome research to clinical diagnoses. However, one of the major challenges of a metagenomic study is the overwhelming non-microbial DNA present in most of the host-derived specimens, which can inundate the microbial signals and reduce the sensitivity of microorganism detection. Various host DNA depletion methods to facilitate metagenomic sequencing have been developed and have received considerable attention in this context. In this review, we present an overview of current host DNA depletion approaches along with explanations of their underlying principles, advantages and disadvantages. We also discuss their applications in laboratory microbiome research and clinical diagnoses and, finally, we envisage the direction of the further perfection of metagenomic sequencing in samples with overabundant host DNA.

## 1. Introduction

**Figure 1:**
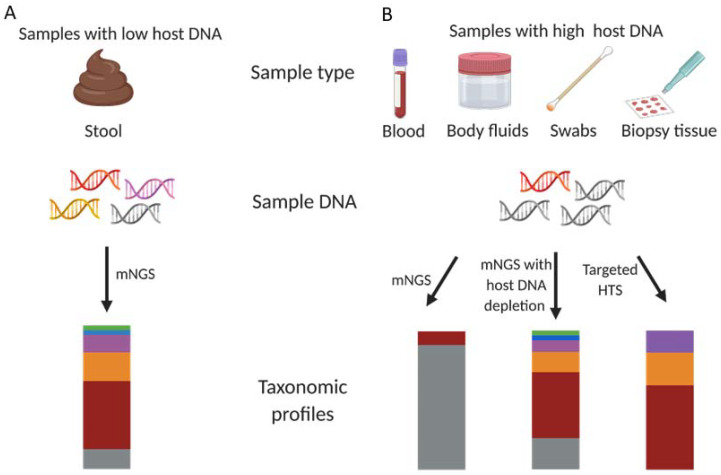
Schematic illustration of untargeted metagenomic sequencing and targeted sequencing in human samples. A variety of human-derived samples can be analysed using untargeted metagenomic sequencing or targeted sequencing. (**A**) For samples with a low amount of host DNA such as faecal samples, a taxonomic profile with a great resolution can be obtained when directly performing untargeted metagenomic sequencing. Gray represents host DNA; red, yellow and purple represent various bacteria; and blue and green represent viruses and archaea, respectively. (**B**) For samples with overabundant human DNA including nasal/oral/skin swabs, body fluids, blood and biopsy tissues, the vast majority of sequencing reads are aligned to the human genome, which can obscure signals from microorganisms when using metagenomic sequencing. As a solution, removal of host DNA before sequencing can improve the resolution of microbial DNA. These samples can also be analysed with targeted sequencing, which can increase the number and proportion of reads of interest in the sequence data although it limits the breadth of microorganisms that can be identified. mNGS, metagenomic next-generation sequencing.

High throughput sequencing (HTS) is widely used in microbiology research and its applications have been rapidly moved from basic research to clinical practice. Any specimen yielding a sufficient amount of nucleic acid can be subjected to an HTS analysis. HTS can either be targeted—that is, enriching certain genes or genomic regions—or untargeted as in metagenomic sequencing [1] (Figure 1). Targeted HTS approaches include universal amplification by a polymerase chain reaction (PCR), multiplex PCR amplification of specific whole genomes [2] and bait capture enrichment [3]. Targeted approaches increase the number and proportion of reads of interest in the sequence data although they limit the breadth of microorganisms that can be identified. For untargeted metagenomic sequencing, the total DNA of all organisms in a sample is sequenced without using any target-specific primers; thus, it covers all genetic information within the sample and allows the detection of all microorganism kingdoms including bacteria, fungi, viruses and parasites [4]. Moreover, metagenomic sequencing can provide a higher taxonomical resolution [5] and enable gene functional analyses such as virulence factors, antibiotic resistance and metabolic networks compared with targeted HTS. These capacities facilitate its great utilisation in microbiome studies.

However, metagenomic sequencing is still challenging to apply to samples with a high host nucleic acid background. Fortunately, a series of methods for depleting host DNA has been developed. This review explores numerous strategies to deplete non-microbial DNA in various specimens. We also discuss potential solutions to constraints and obstacles when using these methodologies in research and clinical diagnoses.

## 2. The Growing Need for Microbial DNA Enrichment Prior to Metagenomic Sequencing

As microbiome studies have progressed, the scope of investigations has gradually shifted from faeces to microbial communities in various regions of the body and from high biomass samples to samples with a low biomass. A human genome is about a thousand times larger than a microbial genome (for example, human genome 3.2 Gb; the bacteria genome is 3.6 Mb on average [6]); therefore, the presence of just a few human cells could completely inundate the DNA components of microorganisms. For example, when the human cell count is over 200 cells per cubic millilitre in the cerebrospinal fluid, this high host DNA background can overwhelm the pathogen signal and decrease the sensitivity of the metagenomic NGS testing [7]. Given that untargeted metagenomic sequencing comprehensively assays all genetic materials regardless of whether they originate from host cells or microorganisms, host DNA has been shown to totally dominate the number of sequencing reads in human skin, vaginal, nasal and oral metagenomes [8]. Whilst the downstream computational filtering of human genome-mapped reads is a common solution, these sequence reads from the human genome can consume unnecessary sequencing space, reduce the overall sensitivity of the assay, obscure microbial differences and even ignore trace pathogen signals. Theoretically, enhancing the sequencing depth (the number of reads generated per specimen [9]) is a potential solution to increase the microorganism-related reads in metagenomic HTS but the huge sequencing costs and analysis time associated with ultra-deep sequencing are far beyond the capability of many laboratories [10] thus limiting the feasibility of the application across a large number of samples.

Collectively, many host-derived samples are deemed to be unsuitable for direct metagenomic sequencing due to their low data yield and high resource requirements. Metagenomic studies of several host-derived samples could benefit from removing the host DNA. This avoids a waste of resources and ensures that only the DNA of interest is sequenced, thereby reducing the amount of sequencing needed to obtain an adequate coverage and depth. More importantly, the removal of host DNA increases the amount and coverage of the microbial reads in metagenomic sequencing, thereby greatly facilitating the subsequent assembly of the reads and the analysis.

## 3. Current Approaches to Host DNA Depletion

Certain differences between the host cell (specifically referring to human beings throughout this review) and the microorganism, including the cellular structure and genomic variances, have been exploited as strategies for microbial DNA enrichment in samples with mixed nucleic acid populations.

### 3.1. Removal of the Host Cells before DNA Extraction

By taking advantage of the disparities in size and density between the microorganisms and eukaryotic host cells, they were filtered with a 5 μm filter, differential centrifugation and a flow cytometry assay, and then were tested to remove the buccal epithelial cells in human saliva [11]. However, no significant difference was observed between any of the host and non-host partitions. In contrast, size-based filtering followed by a deoxyribonuclease (DNase) treatment increased the amount of microbial DNA in sputum samples by 14–33% [12]. These results indicate the critical impact of extracellular DNA originating from host cells when physically separating the host cells and microorganisms. A combination of physical separation with DNase digestion can achieve a greater efficiency in enrichment. On the other hand, enriching virus-like particles by physical methods is a routine pre-treatment of viral metagenomic sequencing. Filtration is frequently used as the first step to purify virus-like particles because viruses (20–500 nm) are much smaller than host cells; hence, viruses can readily be separated from the host cells by physical methods. Typically, filters with pore sizes of 0.2 μm and 0.45 μm are used [13]. Size-based filtering followed by a DNase treatment is also capable of virus enrichment [14]. Due to the compact nature of virions, it is also possible to enrich the amount of virus-derived nucleic acids in samples using density gradient centrifugation (e.g., sucrose, caesium chloride, polyethylene glycol).

**Figure 2:**
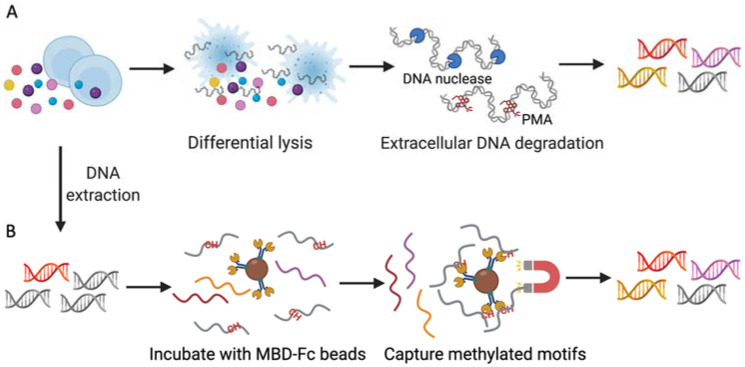
Workflow of typical host DNA depletion approaches. Before metagenomic sequencing, human DNA can be removed by different approaches. (**A**) The mainstream pre-extraction method to remove host DNA is first treating human cells with a selective lysis buffer followed by DNA nuclease or PMA treatment. (**B**) The most commonly used post-extraction methods take advantage of the disparity of the cytosine methylation frequency between eukaryotic and prokaryotic DNA. MBD-Fc-bound magnetic beads can capture methylated human DNA sequences, leaving the unmethylated motifs for downstream library preparation. MBD, methyl-CpG binding domain; HTS, high throughput sequencing.

A cell wall can be found in the majority of bacteria and fungi and is substantially harder than the plasma membrane of mammalian cells. For viruses, although they have no cell wall, a protein capsid of a naked virus is sufficient to allow viruses to have a resistance against many disinfectants. In this context, a mild lysis buffer could be used to selectively lyse the plasma membrane of the host cells without damaging the microorganisms. The released DNA from the lysed host cells is then degraded by DNase, leaving intact microorganisms for a downstream extraction (Figure 2).

A variety of reagents including sterile water, saponin (a non-ionic detergent belonging to the group of glycosides forms), Triton X-100, Tween 20 and zwitterionic detergent have been assessed for their ability to selectively lyse human cells [15,16,17]. Among them, saponin has been the most widely used lysis reagent of mammalian cells owing to its high lysis efficiency on host cells with minimised effects on bacteria, fungi and even a non-enveloped “naked” virus [15]. An optimised two-step lysis protocol, in which treating the sample with saponin followed by an osmotic shock using double-distilled water, can further boost its efficiency [18,19,20].

Once the plasma membrane is destroyed by the lysis reagent, the DNA in the host cells is released. DNase I is commonly employed to degrade extracellular DNA [12,21], yet its activity depends closely on the choice of appropriate reaction buffers and could be limited by the presence of selective lysis reagents. Therefore, Benzonase nuclease has become an optimal approach for host DNA depletion due to its wide range of operating conditions and exceptionally high specificity, which allows the cleavage of exposed nucleic acid into 3–5 bases in length [22,23,24,25,26]. For samples already lysed by saponin and osmotic shock, using heat-labile salt active nuclease (HL-SAN; it has optimal activity at a high salt concentration) can achieve a 1000-fold reduction in the amount of human DNA [20,27]. Propidium monoazide (PMA) was found to have multiple advantages over conventional enzymatic degradation including a lower cost as well as being less time-consuming and having fewer sample processing steps. PMA is a cell membrane-impermeable DNA intercalator that can covalently modify DNA in the dark so that the DNA cannot be further amplified; the remaining PMA can be inactivated by light exposure [28]. These unique features make PMA an alternative to a DNA nuclease without the necessity of including any washing steps that could result in a loss of DNA.

Several commercially available protocols (Table 1) have been developed with applications of a series of lysis reagents and nucleases [11,29,30,31,32,33,34,35]. Generally, all these kits (or their modified protocols) exhibit a good efficiency in host DNA depletion and can increase the proportion of microbial DNA in a variety of sample types including blood [36], skin [31,37], bronchoalveolar lavage fluid [22] and tissues [32]. However, for samples with trace amounts of a microbial content, degrading the host nucleic acid using these approaches can lead to insufficient amounts of DNA for the sequencing library preparation. To ensure the amount of microbial DNA, whole genome amplification (WGA) by multiple displacement amplification can be used to amplify the raw DNA from nanograms to micrograms, providing enough DNA for the library preparation and sequencing. In a study with samples of sonicated fluids from a resected arthroplasty component, WGA was adopted to obtain a sufficient quantity of DNA for the library preparation after human DNA depletion by a MolYsis^TM^ Basic kit (see Table 1) [30,38]. However, WGA can introduce an amplification bias [39] and any microbial or cross-contamination among samples could also be magnified [40]. To solve these issues, WGA performed in sub-nanolitre droplets could significantly reduce the amplification bias and contamination [41,42] as this technique reduces competition for the primers and polymerases among the DNA fragments via the partitioning of the template DNA into tiny individual spaces. For example, a study by Shi et al. [12] utilised a microfluidic chip to separate microorganisms in samples and amplify their DNA in droplets to efficiently recover the microbial genome with a markedly reduced amplification bias.

### 3.2. Separating the Microbial DNA from the Host Background

The recent development of highly multiplexed sequence capture approaches has enabled the enrichment of DNA from hundreds of known viruses and bacteria by selectively amplifying their DNA sequences as capture tools. These probes can be immobilised on solid carriers or biotinylated for sequence-specific hybridisation with the target DNA. Of note, an intrinsic limitation is that a depletion by a microorganism-specific hybridisation capture is not a viable option when the DNA sequence of interest is unknown in advance because the microbial DNA sequence has to be determined to enable the design of specific probes. Alternatively, probes can be designed to capture the host genome for the purpose of studying entire microbial communities harbouring a mixture of microorganisms with known or unknown sequences [48]. However, this hybridisation-based clean-up approach is inefficient due to the large size and complexity of the human genome.

Both prokaryotes and eukaryotes undergo methylation as their major epigenetic event but microbial and human epigenetics have distinct characteristics and functions. For higher eukaryotes, 5-methylcytosine is the dominant form of methylated DNA [49]. In humans, methylated cytosine occurs predominantly in a context of CpG dinucleotide with an estimated frequency of 60% to 90% [50]. The NEBNext Microbiome DNA Enrichment Kit (Table 1) employs a methyl-CpG binding protein domain to pull down vertebrate DNA based on this methylation pattern in eukaryotes. In comparison, a derivative of human CXXC finger protein-1 with a specific affinity to non-methylated CpG dinucleotides [51] was adopted for prokaryotic DNA isolation in a LOOXSTER^®^ enrichment kit [52]. Moreover, several cytosine methylation-dependent endonucleases such as MspJI [53], HpaII and McrB [54] have been reported as feasible tools for microbial DNA enrichment.

One of the most studied functions of DNA methylation in prokaryotes is as a component of the restriction-modification system. Restriction enzymes in prokaryotes can cleave foreign DNA in this system whereas DNA methylation protects the prokaryotic genome from destruction [55]. N6-methyladenine modification is widespread in prokaryotes but has been rarely reported in eukaryotic genomes [56]. DpnI is a methyl-directed restriction endonuclease that restricts DNA only when it is methylated on adenine residues within the GATC sequence [57]. A DpnI-mediated DNA enrichment strategy was introduced in which only bacteria but not human DNA could specifically bind to DpnI immobilised on magnetic beads [58]. This method enabled more than a 100-fold enrichment of the prokaryotic genomes present at 1/10,000 of the level of human DNA.

### 3.3. Limitation and Controversy

In general, the selective lysis of human cells followed by the degradation of background DNA is shown to be effective in reducing the host DNA. However, the most obvious downside of these methods is a biased recovery among the microbial species due to their unequal sensitivities to the lysing conditions. For example, *Mycoplasma* spp. and parasites are more likely to be destroyed by a selective lysis buffer, leading to a low recovery in sequencing. Freezing and thawing can also potentially disrupt the microorganisms, thus emphasising the preferential use of fresh samples and the avoidance of having multiple freeze–thaw cycles. Moreover, these protocols involve multiple steps of lysis and centrifugation that inevitably cause DNA loss, thus limiting the potential of low biomass samples to be successfully processed [15,59].

The methylomes of only a few bacterial species have been well defined; the methylomes of protists, fungi and viruses are even less characterised. Several bacteria exhibit a similar methylation pattern with the human genome; for instance, *Helicobacter pylori* exhibits a high density of 5 mC modification [60], which is similar to human DNA methylation. Fungi also display a large portion of 5 mC content in their genomes [61] as well as DNA viruses, which demonstrate that complex cytosine methylation is involved in the genome replication state and host environment [62]. However, the selective enrichment of microbial sequences using DNA binding proteins or methylation-dependent endonucleases greatly depends on the methylation state of the target genomes, resulting in an unequal recovery of the microbial reads and distorting the ratio of the different microorganism lineages. For example, although most bacterial genomes could be increased 70- to 200-fold using HpaII, a failure in enriching *Borrelia burgdorferi* has been reported [54]. Thus, methylation-based isolation methods have limited the types of microbes that can be enriched.

Of note, comparing the efficacy of approaches across different samples could be unjustified. The efficacy of an approach could be varied among different type of samples, depending on the original ratio of non-host DNA to host DNA. For example, if the proportion of microbial DNA in a sample is 10%, it can only be enriched 10-fold at most to reach the maximal 100%; if the proportion of microbial DNA is lower than 1%, it can be enriched 100-fold to reach the maximum. The disparities in analyses and sample properties can also lead to controversial results. For example, Marotz et al. concluded that an osmotic lysis followed by a PMA treatment in saliva samples was promising to remove host-derived sequencing reads with only a small taxonomic bias [11]. In contrast, Ganda et al. argued that this method not only decreased the host DNA but also reduced the bacterial DNA extracted from a bovine milk sample in a dose-dependent manner [35]. One explanation is the difference in approaches to assess the enrichment efficacy between these two studies. The first study used shotgun sequencing to acquire reads from various organisms at a relative proportion whereas the latter was based on qPCR to quantify the exact amount of bacterial DNA. A microbiome study also reported that a PMA treatment could not impact on the human DNA proportion in a sputum sample due to its complexity and viscosity compared with saliva [24]. Nevertheless, despite these limitations, current host DNA depletion methods enable the characterisation of microbial profiles in samples dominated by human DNA and these approaches are being increasingly used in clinical metagenomics.

## 4. Application in Microbiome Research and Clinical Metagenomics

The majority of current human microbiome research focuses on characterising the microbial profile and its association with host gene functions in health and disease. As previously stated, several studies have succeeded in depleting the host DNA in various human samples. For instance, an osmotic lysis followed by a PMA treatment on human saliva could increase the amount of microbial sequencing reads from 1% to 89% [11]. Moreover, in the field of metagenomic research on non-human hosts, the host DNA depletion is also critical. For example, in a resistome study of the milk production environment, depleting the host DNA before extraction was proven to be an efficient approach to remove the bovine reads, thereby facilitating the identification of the antimicrobial resistance gene [33].

However, not all specimens are suitable to be subjected to host DNA depletion approaches. The major reason is that the amount of depleted human DNA is not enough to effectively change the relative abundance of the reads assigned to the microbial genome in metagenomic HTS studies [35]. In actual practice, the final proportion of the microbial DNA after host DNA depletion deserves greater concern rather than focusing on the fold of the reduction in the host DNA. In a few cases, even if the host DNA is largely eliminated, the ratio of microbial DNA to human DNA is still too low for a metagenomic analysis. For example, our unpublished data showed that the bacteria-to-human DNA ratio in a gastric biopsy was extremely low at 1:1,000,000. Even if the human DNA could be reduced 1000-fold by host DNA depletion, the ratio of the bacteria-to-human DNA was still about 1:1000 or even lower. This indicated that if 15 GB of raw data was obtained from sequencing, only 15 MB of the data belonged to bacterial DNA, which obviously demonstrated that very limited information could be provided. Such an inadequacy could also be the major reason why amplicon-targeted sequencing is predominantly used in microbiome research with low biomass samples.

Clinical metagenomics require a broad identification of known and uncharacterised pathogens, thereby providing genomic information for evolutionary tracing, mutation discovery and drug resistance characterisation [63,64]. The primary goals of clinical metagenomics differ from those of basic research; a microbiome study often focuses on the relationship between microorganisms and disease, necessitating more microorganism-related data and an unbiased presentation of diverse microbial populations whereas clinical metagenomics pay more attention to microbial detection—the presence or absence of pathogens in a more time- and cost-dependent manner than basic research. For untargeted metagenomic sequencing, where a broad spectrum of pathogens can be identified in a single assay [65], the efficient removal of background human genetic material could concentrate the pathogen DNA to increase the sensitivity of the detection. Therefore, host DNA depletion (Table 2) is more widely used in clinical metagenomics compared with basic microbiome research.

Most clinical samples are processed promptly to avoid the release of both the host and microbial DNA from the freeze–thaw cycle. Therefore, differential centrifugation [80,81] and a pre-lysis followed by a DNA nuclease [20,66] are reliable methods to minimise the amount of host DNA in fresh samples. Unlike mock samples cultured in the laboratory, microorganisms in clinical specimens may have already been harmed by host immune cells or antibiotic treatments before sampling or damaged during the transportation and handling of the sample. These events inevitably result in a certain extent of release of cell-free DNA from pathogens that could be subsequently lost during selective lysis protocols. In comparison, although a methylated-CpG capture-based strategy is less efficient than a selective lysis [30] with a reported biased recovery toward several microbes such as *Neisseria flavescens* [46], such a strategy is capable of retaining cell-free DNA from dead organisms to avoid DNA loss, as observed when using a selective lysis [68,79]. Combining size- or density-based separation methods with a methylated CpG capture strategy could increase the proportion of the host DNA depletion whilst retaining the cell-free microbial DNA.

To ensure accuracy and avoid false-positive results, a threshold should be established prior to the detection [65]; a species or genus should only be considered to be “detected” if it meets the threshold otherwise it should be reported as “not detected”. In this context, reporting the presence or absence of a pathogen does not require a large amount of sequencing reads from the HTS testing. Therefore, for a low biomass sample, even the ratio of pathogen-to-human DNA would be about 1:1000 or less; it could still be considered to be positive as long as its signal exceeded the threshold. Therefore, the host DNA depletion could be robustly applied to low biomass samples in clinical metagenomics as the increased amount of the pathogen reads in the patients could simultaneously enhance the sensitivity of the diagnosis. Emerging data have suggested that the host DNA depletion in clinical metagenomics facilitates an improvement in the diagnosis sensitivity as well as the discovery and identification of potential pathogens and gene features [20,68,71]. These approaches are still being challenged by the reproducibility of the results and a potential contamination in the reagents [82]. Proper consideration of these issues is necessary to enable the future application of host DNA depletion in clinical metagenomics.

## 5. New Strategies to Facilitate Metagenomic Sequencing in Samples with Overabundant Host DNA

**Figure 3:**
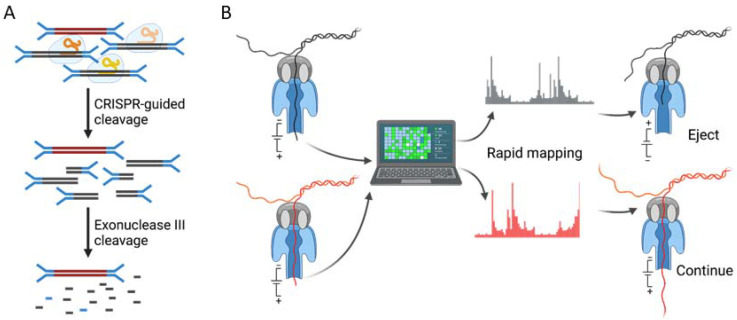
Illustration of strategies for removing unwanted high abundance DNA. (**A**) Sequencing library with Y-shape adapters contacts with a plurality of protein-guide RNA (gRNA) complexes in CRISPR/Cas9 system wherein gRNAs are complementary to the targeted human sequences to allow cleavage. Cleaved host DNA is then degraded by exonuclease III from blunt-ends cleaved by Cas9, leaving other sequences intact for subsequent amplification and sequencing [83]. (**B**) Using a nanopore device and computational approaches, individual double-strand DNA molecules can be selectively sequenced. When the DNA strand is sequenced, its current signal can be rapidly classified with or without base-calling. If the molecule is mapped to the pre-set reference genome such as a human genome, these reads would then be ejected from pores in real-time by revering the voltage polarity; otherwise, the sequencing would continue. Figures created using BioRender (https://biorender.com, accessed on 3 February 2022).

In studies of DNA or RNA, a large proportion of nucleic acid molecules that are irrelevant to the question at hand are frequently encountered. Recently, several novel strategies for removing unwanted nucleic acid have been reported that may help to eliminate the host DNA in metagenomic sequencing (Figure 3).

### 5.1. The Removal of Unwanted High Abundance Species in Sequencing Libraries

The clustered regularly interspaced short palindromic repeats (CRISPR)/CRISPR-associated protein 9 (Cas9) system provides opportunities for depleting the targeted nucleic acid sequences in a sample (Figure 3). It works well in RNA sequencing to remove mitochondrial rRNA, which is the most abundant sequence in cerebral fluid-derived RNA samples [84]. However, to date, there is no comparable efficiency for using such an approach to deplete the host DNA as targeting the entire human DNA genome is impractical due to its requirement of a substantially high cost but with a low efficiency. One potential solution to improve the performance is to design guide RNA-targeting multicopy sequences such as primate-specific Alu elements, which comprise 11% of the entire human genome [85]. On the other hand, a combination of a CRISPR/Cas9 cleavage with long read sequencing can also be a feasible solution. Given that only one Cas9 cleaving site is required per each long DNA fragment, unwanted sequences could be effectively removed.

### 5.2. Selective Sequencing

The Oxford Nanopore MinION is a portable real-time sequencing device that functions by sensing the change in the current flow through a nanopore when DNA passes through. Nanopore sequencing has the significant advantage of providing real-time data and analyses compared with the conventional Illumina platform, thereby allowing a fast turnaround time of clinical results. Another critical advantage of nanopore sequencing is its unique capability of conducting selective sequencing (Figure 3). Individual molecules can be selectively sequenced (ReadUntil) using only computational methods by allowing nanopore devices to selectively eject reads from the pores in real-time through reversing the voltage polarity across the specified pores for a short period of time (~0.1 s) [86]. However, this requires the rapid classification of the current signal from the first part of a read to determine whether the molecule should be sequenced or removed and replaced with a new molecule. Recently, an open-source mapper named UNCALLED [87] (https://github.com/skovaka/UNCALLED, accessed on 3 February 2022) has been established that can rapidly match the streaming nanopore current signals to a reference sequence without base-calling. Another recently developed toolkit, Readfish [88] (https://www.github.com/looselab/readfish, accessed on 3 February 2022), requires a sufficiently fast base-caller but can also design and control selective sequencing procedures by removing the need to have complex signal mapping algorithms. To date, selective sequencing has been used in a variety of studies including the depletion of known bacterial genomes within a metagenomic community, the enrichment of certain specific human genes associated with hereditary cancers and the enrichment of low abundance genomes from samples with mixed populations [87,88]. Although selective sequencing is yet to be implemented in depleting the whole human genome, this approach could theoretically be an attractive alternative for selectively enriching microbial DNA in human background DNA without a pre-sequencing enrichment during the sample preparation.

Nevertheless, there are always gaps between a newly established method and its popularisation. CRISPR/Cas9 system-assisted methods and selective sequencing by Oxford Nanopore MinION are currently conceptual applications for host DNA depletion; hence, further validation studies are needed to assess whether these strategies could be promising to enrich the microbial reads in samples with overabundant host DNA without a bias.

## 6. Conclusions and Future Perspectives

A range of host DNA depletion and microbial DNA enrichment methods for metagenomic sequencing have been developed and evaluated in samples with varying characteristics. Among all approaches, one consistent conclusion is that host DNA depletion improves the efficiency and sensitivity of metagenomic sequencing. The main controversy among these studies is the variation in performance across the methods and sample types, highlighting the need for an individual assessment of each host depletion method to determine its desired sample type. Numerous factors should be considered when choosing an optimal method including the sample type (e.g., solid tissues or liquid samples; fresh or frozen), microbial load, pathogens of interest and budget.

Identifying the difference between the host and microbial genome can theoretically broaden possible strategies to achieve a microbial DNA enrichment. Despite the fact that most of these new methods are not commercially available, they are still worth optimising given their promising initial results. A few novel strategies have aimed to eliminate high abundance DNA populations during the library preparation or sequencing steps to achieve a human DNA depletion. Overall, continuous efforts are needed to validate new approaches when using metagenomics to study microbiomes in samples with large amounts of DNA derived from a human host.

## Figures and Tables

**Table 1:** Commercial kits for microorganism enrichment.

Kit	Principle	Pros	Cons	Hands-On Time Per Sample	Cost Per Sample (USD)	Ref.
QIAamp DNA Microbiome (Qiagen, Hilden, Germany)	Lysis of host cell by saponin, degrade extracellular DNA with Benzonase nuclease	Ultra-clean columns to minimise contamination risk	Requires fresh sample	160 min	13	[43]
MolYsis™ Complete/Ultra-Deep Microbiome Prep (Molzym, Bremen, Germany)	Chaotropic lysis of host cell, degrade extracellular DNA with MolDNase	Applicable for body fluids, tissue and swab samples. Enrichment of bacterial and fungal DNA	Fresh sample is recommended	120 min	11	[44]
HostZERO Microbial DNA Kit (Zymo Research, Irvine, CA, USA)	Lysis of host cell, degrade extracellular DNA with microbial selection enzyme	Protocols for both tissue and liquid samples are provided	Requires intact (living) bacteria cells	30 min	10	[45]
NEBNext Microbiome DNA Enrichment (New England BioLabs, Ipswich, MA, USA)	Capture methylated host DNA	Can retain cell-free DNA from dead organisms to avoid DNA loss	Requires high molecular weight intact DNA. Bias to high CpG-methylated microbes	30 min *	39 *	[46]
LOOXSTER Enrichment Kit (Analytik Jena GmbH, Jena, Germany)	Capture non-methylated CpG dinucleotides	Can retain cell-free DNA from dead organisms to avoid DNA loss	Requires high molecular weight intact DNA. Bias to high CpG-methylated microbes	75 min *	34 *	[47]

*: DNA extraction step is excluded.

**Table 2:** Case examples of host DNA depletion in clinical metagenomics in the last five years.

Sample Type	Potential Clinical Indication	Sample Size	Depletion Method	Sequencing Platform	Reads Number	Ref.
Cerebrospinal fluid	Infectious aetiology identification	13	Selective lysis by a bead-beater tissue homogeniser followed by a Benzonase nuclease treatment	Ion Torrent PGM	N/A	[23]
Prosthetic joint sonicate fluid	Pathogen identification	408	MolYsis basic kit	Illumina HiSeq	2.8 million, mean	[38]
Urine	Pathogen identification	10	Differential centrifugation and MolYsis kit	MinION	0.026 million, median	[66]
Urine	Antimicrobial resistance marker identification	13	NEBNext microbiome kit	Ion Torrent PGM	N/A	[67]
Sputum	Pathogen detection	6	Microfluidic separation followed by DNase digestion	Illumina HiSeq	36.3 million, mean	[12]
Sputum, bronchoalveolar lavage and endotracheal aspirates	Diagnosis of known and unknown infections	40	Saponin-based differential lysis followed by HL-SAN DNase digestion	MinION	0.041 million, mean	[20]
Cerebrospinal fluid	Diagnosis of known and unknown infections	95	NEB Microbiome Enrichment Kit	Illumina HiSeq	5~10 million	[68]
Endotracheal aspirates	Pathogen identification	22	Saponin-based differential lysis followed by HL-SAN DNase digestion	MinION	6628, median	[69]
Synovial fluid	Pathogen detection	168	MolYsis basic kit	Illumina HiSeq	30 million, mean	[70]
Bone and joint infectious tissue	Pathogen detection and antibiotic susceptibility prediction	24	Ultra-Deep Microbiome Prep kit	Illumina HiSeq	20 million, mean	[71]
Valve tissue	Pathogen identification	1	Ultra-Deep Microbiome Prep kit	Illumina MiSeq	1.4 million, mean	[72]
Hepatic tissue	Diagnosis of unknown infections	1	Ultra-Deep Microbiome Prep kit	Illumina MiSeq	1.1 million, mean	[73]
Blood culture bottles inoculated with prosthetic joint tissue	Pathogen identification	9	MolYsis basic kit	Illumina MiSeq	10.3 million, mean	[74]
Blood	Pathogen detection	8	MolYsis complete kit and WGA	Illumina HiSeq	27.5 million, mean	[75]
Whole blood	Diagnosis of infection	101	MolYsis complete kit	Ion Torrent	N/A	[76]
Sputum	*M. tuberculosis* detection and antibiotic susceptibility prediction	40	MolYsis basic kit	Illumina MiSeq and MinION	3.6 million, mean	[77]
Prosthetic joint sonication fluid	Diagnosis of prosthetic joint infections	97	A 5 μm pore size filter	Illumina MiSeq	N/A	[78]
Urine	Pathogen detection and antimicrobial susceptibility prediction	40	NEB Microbiome Enrichment Kit	Ion Proton	N/A	[79]

N/A: data is not publicly available for analysis.

## Data Availability

Data are available within the article.
